# Depression as a Glial-Based Synaptic Dysfunction

**DOI:** 10.3389/fncel.2015.00521

**Published:** 2016-01-22

**Authors:** Daniel Rial, Cristina Lemos, Helena Pinheiro, Joana M. Duarte, Francisco Q. Gonçalves, Joana I. Real, Rui D. Prediger, Nélio Gonçalves, Catarina A. Gomes, Paula M. Canas, Paula Agostinho, Rodrigo A. Cunha

**Affiliations:** ^1^CNC - Center for Neuroscience and Cell Biology, University of CoimbraCoimbra, Portugal; ^2^Departamento de Farmacologia, Universidade Federal de Santa Catarina, Florianópolis, SCBrazil; ^3^Faculty of Medicine, University of CoimbraCoimbra, Portugal

**Keywords:** depression, synapse, astrocytes, microglia, purines

## Abstract

Recent studies combining pharmacological, behavioral, electrophysiological and molecular approaches indicate that depression results from maladaptive neuroplastic processes occurring in defined frontolimbic circuits responsible for emotional processing such as the prefrontal cortex, hippocampus, amygdala and ventral striatum. However, the exact mechanisms controlling synaptic plasticity that are disrupted to trigger depressive conditions have not been elucidated. Since glial cells (astrocytes and microglia) tightly and dynamically interact with synapses, engaging a bi-directional communication critical for the processing of synaptic information, we now revisit the role of glial cells in the etiology of depression focusing on a dysfunction of the “quad-partite” synapse. This interest is supported by the observations that depressive-like conditions are associated with a decreased density and hypofunction of astrocytes and with an increased microglia “activation” in frontolimbic regions, which is expected to contribute for the synaptic dysfunction present in depression. Furthermore, the traditional culprits of depression (glucocorticoids, biogenic amines, brain-derived neurotrophic factor, BDNF) affect glia functioning, whereas antidepressant treatments (serotonin-selective reuptake inhibitors, SSRIs, electroshocks, deep brain stimulation) recover glia functioning. In this context of a quad-partite synapse, systems modulating glia-synapse bidirectional communication—such as the purinergic neuromodulation system operated by adenosine 5′-triphosphate (ATP) and adenosine—emerge as promising candidates to “re-normalize” synaptic function by combining direct synaptic effects with an ability to also control astrocyte and microglia function. This proposed triple action of purines to control aberrant synaptic function illustrates the rationale to consider the interference with glia dysfunction as a mechanism of action driving the design of future pharmacological tools to manage depression.

## Introduction

Depression is the neuropsychiatric disorder with higher incidence worldwide, representing a major socio-economical burden (Kessler et al., 2003). Depressive conditions display heterogeneous presentations and are defined clinically based on different affective symptoms (sadness, desperation, apathy, anhedonia, sensation of discomfort) that decrease interest in daily activities (Ferrari et al., 2013). Understanding the causes and neurobiological basis of depression remains a challenge, probably due to the lack of faithful animal models (Berton et al., 2012). The relation between cumulative stress and the incidence of depression (de Kloet et al., 2005) and the prevalence of depression in suicide completers (Coryell and Young, 2005), provide two windows of opportunities to indirectly study the neurobiological basis of depression using chronically stressed animals and brain samples from suicide completers.

## Purported Biological Basis of Depression

Depressive conditions have traditionally been considered to involve a deregulated hypothalamic-pituitary-adrenal (HPA) axis, leading to an aberrant impact of sustained increased levels of glucocorticoids (Sousa et al., 2008). However, recent studies in adrenalectomized animals (thus blunting the main source of glucocorticoids) revealed an impact of ghrelin rather than glucocorticoids in formatting the emotional disturbances associated with repeated stress (Meyer et al., 2014).

Another lead for the neurobiology of depression is based on the clinical use of serotonin-selective reuptake inhibitors (SSRIs) and tricyclic antidepressants (TCAs) to manage depression, which hints at the dysfunction of the neuromodulation by serotonin and other biogenic amines in depression (Krishnan and Nestler, 2008). However, these drugs have a slow onset of action (over 2 weeks) and are effective in only* circa* 50% of patients, leaving open the possibility that they may influence different targets apart normalizing the levels of biogenic amines.

Neurotrophins and in particular brain-derived neurotrophic factor (BDNF) have also been linked to depression, based on the ability of BDNF to reactivate neuronal plasticity and on the association between serum BDNF levels and depression (Castrén, 2014). This should be cautiously evaluated since the plasma levels of substances might not reflect their brain levels and there is no clear association between depression and different polymorphisms of the *bdnf* gene (Gyekis et al., 2013). Also, excessive BDNF-induced plasticity can actually trigger an aberrant hyper-plasticity, as heralded by the key pathogenic role of BDNF in neuropathic pain (Trang et al., 2012).

## Synaptic Dysfunction in Depression

A major breakthrough in understanding depression was the observation that sub-anesthetic doses of ketamine, an NMDA receptor antagonist, can revert rapidly (within 90 min) symptoms of depression, with a long-lasting antidepressant effect (2 weeks; Berman et al., 2000; Zarate et al., 2006). Since NMDA receptors are the main switches to trigger synaptic plasticity (both long-term potentiation, LTP, and depression, LTD), this indicates that an abnormal plasticity at glutamatergic synapses underlies the expression of depressive symptoms (Duman and Aghajanian, 2012); indeed, chronically-stressed rodents display abnormal patterns of synaptic plasticity (both LTP and/or LTD) in brain areas involved in emotional processing, namely amygdala, ventral striatum, hippocampus and prefrontal cortex (Krishnan and Nestler, 2008). Animal studies also allowed identifying the molecular mechanisms of the antidepressant effect of ketamine, which involves the antagonism of NMDA (2B) receptors and the preservation of dendritic morphology and AMPA receptor trafficking through an mTOR pathway in the prefrontal cortex (Li et al., 2010). This joins other observations showing that riluzole and antagonists of types 2/3 or 5 metabotropic glutamate receptors, which control glutamatergic transmission, also display robust antidepressant effects (Machado-Vieira et al., 2009; Pilc et al., 2013). Altogether these observations support the hypothesis that depression results from the disruption of mechanisms controlling synaptic plasticity in afflicted regions (Duman and Aghajanian, 2012).

This de-regulation seems to translate into a destabilization and loss of synaptic connections. Indeed, repeated stress triggers a reduction of dendritic complexity in prefrontocortical and hippocampal neurons (Magariños et al., 1997; Sousa et al., 2000; Radley et al., 2006) and a selective loss of markers of excitatory synapses (Gilabert-Juan et al., 2012; Tzanoulinou et al., 2014; Kaster et al., 2015), which recover upon alleviation of “depressive”-like symptoms using SSRIs, exercise or enriched environment (Li et al., 2010, 2011; McEwen et al., 2012). Post-mortem brain samples of depressed patients also revealed a reduction in the size rather than number of prefrontocortical and hippocampal pyramidal neurons (Rajkowska et al., 1999; Stockmeier et al., 2004) accompanied by a decreased number of synaptic contacts (Kang et al., 2012). As occurs in stressed rodents, synaptic markers in frontolimbic area are also altered in patients with major depressive disorder (Feyissa et al., 2009; Zhao et al., 2012; Duric et al., 2013).

This pivotal role of the disruption of mechanisms controlling synaptic plasticity for the expression of depressive symptoms also has the attractive feature to allow bridging the different traditional explanations for the emergence of depression. In fact, glucocorticoids are well established to affect synaptic plasticity and to contribute for synaptic atrophy in several brain regions (Sousa et al., 2008). Likewise, BDNF is well recognized as a bolster of synaptic plasticity (Gray et al., 2013) and different biogenic amines, such as serotonin (Lesch and Waider, 2012), noradrenaline (Marzo et al., 2009) and dopamine (Tritsch and Sabatini, 2012) impact on synaptic plasticity in cortical regions.

However, although the pivotal role of aberrant synaptic plasticity successfully integrates different findings derived from patients and animal models of depression, it still fails to provide an explanation for the etiology of depression. This review proposes to focus on the emerging concept of the quad-partite synapse (Schafer et al., 2013), integrating both astrocytes and microglia as critical pillars of synaptic plasticity, to address the possible relevance of a mis-communication between glia and synapses, as a possible basis of depression.

## The “Quad-Partite” SYNAPSE

Astrocytes were long considered as morphological and metabolic support cells, as testified by their importance in the synthesis (Rose et al., 2013) and re-uptake of glutamate (Asztely et al., 1997; Arnth-Jensen et al., 2002), in buffering extracellular K^+^ to control neuronal excitability (Wallraff et al., 2006), in neurovascular coupling (Viswanathan and Freeman, 2007; Petzold et al., 2008; Figley and Stroman, 2011) and transport (Rouach et al., 2008) and delivery of nutrients to active synapses (Magistretti et al., 1999; Pellerin et al., 2007). This concept has actually evolved to recognize astrocytes as dynamic players engaged in a bi-directional communication with synapses and able to actually format synaptic function with impact on the expression of behavior (Achour and Pascual, 2010; Allen, 2014; Oliveira et al., 2015). This interplay between synapses and astrocytes is so tight that it is difficult to disentangle if a synaptic dysfunction results from intrinsic modifications of neurons or from astrocytic modifications (Agostinho et al., 2010; Sanacora and Banasr, 2013; Crunelli et al., 2015; Verkhratsky et al., 2015).

Probably the first experimental support of an ability of astrocytes to respond to synaptic activity was provided by observations that astrocytes respond to glutamate by triggering a directional long-distance response, typified by a wave of variation of intracellular calcium (Cornell-Bell et al., 1990). The inter-astrocytic transport of calcium waves is possible thanks to the organized formation of a syncytium through different connexins pores, that form an alphabet still to be fully deciphered to understand this long-range directional communication of information through astrocytes (Wallraff et al., 2004; Theis and Giaume, 2012; Decrock et al., 2015). Astrocytes respond not only to glutamate, but to most neurotransmitters and neuromodulators, such as GABA, noradrenaline, acetylcholine or adenosine 5′-triphosphate (ATP; Volterra and Meldolesi, 2005; Haydon and Carmignoto, 2006). Astrocytic calcium waves can feedback to influence neuronal responses (Nedergaard, 1994; Parpura et al., 1994) and control synaptic strength (Jourdain et al., 2007; Perea and Araque, 2007) through the release of different mediators such as glutamate, ATP, D-serine, NO, neurotrophins, prostaglandins or cytokines to name a few (Volterra and Meldolesi, 2005; Haydon and Carmignoto, 2006). Several of these mediators are released from astrocytes in a vesicular manner, with SNARE complexes similar, but with some differences to neurons (e.g., synaptobrevin 2), able to sustain a quantal release (Bezzi et al., 2004; Pangrsic et al., 2007). The relevance of this astrocytic vesicular apparatus to control synaptic function is re-enforced by the observation that astrocytic processes enwrap synapses in a spatially organized manner, with a single astrocyte wrapping from 300 (in rodents) up to 90,000 (in humans) synapses (Bushong et al., 2002; Ogata and Kosaka, 2002; Oberheim et al., 2006) and this association of astrocytes with synapses was found to be an experience-dependent dynamic process (Genoud et al., 2006; Haber et al., 2006). This prompted the concept of the tri-partite synapse to recognize the importance of the astrocytic network as a new level of integration of information in neuronal networks (Araque et al., 1999, 2014; Halassa et al., 2007). Accordingly, synaptic plasticity processes are controlled by different gliotransmitters such as D-serine (Yang et al., 2003; Panatier et al., 2006; Henneberger et al., 2010), glutamate (Fellin et al., 2004), ATP (Koizumi et al., 2003; Zhang et al., 2003; Pankratov and Lalo, 2015) or adenosine (Newman, 2003; Pascual et al., 2005; Serrano et al., 2006) or by controlling glutamate clearance (Diamond, 2001; Tsvetkov et al., 2004; Omrani et al., 2009; Murphy-Royal et al., 2015). Accordingly, astrocytic function critically affects integrated brain responses such as sleep, mood or memory (Banasr and Duman, 2008; Halassa et al., 2009; Suzuki et al., 2011; Lima et al., 2014; Perea et al., 2014; Matos et al., 2015).

The addition of microglia to the number of synaptic players is more recent. Microglia coordinate brain innate immunity, displaying features characteristic of immune cells able to rapidly expand their population, to chemotaxically migrate to sites of injury and to trigger and sustain inflammatory responses through their chemokine and cytokine repertoire (Lynch, 2009; Kettenmann et al., 2011). Traditionally, microglia were considered to be “resting”, becoming “activated” upon allostatic changes to coordinate immune-like responses (Perry and Gordon, 1988). Several studies revealed that the purported “resting”-state of microglia actually corresponds to an active surveying state, where microglia phylopodia dynamically interact with neurons and astrocytes with a regulatory and supportive role critical for brain homeostasis (Raivich, 2005; Hanisch and Kettenmann, 2007; Wake et al., 2013; Cherry et al., 2014). In particular, microglia dynamically interact with synapses in an activity-dependent manner (Biber et al., 2007; Kettenmann et al., 2013; Wake et al., 2013), to such as extent that the concept of a quad-partite synapse has been forwarded (Schafer et al., 2013). In fact, microglia are equipped with receptors for neurotransmitters (Pocock and Kettenmann, 2007), and excitatory transmission increases whereas inhibitory transmission decreases microglial processes dynamic (Fontainhas et al., 2011; Wong et al., 2011). Conversely, microglia can affect both excitatory and inhibitory transmission (Tsuda et al., 2003; Pascual et al., 2012) through the release of a variety of signals ranging from chemokines (Schafer et al., 2012), cytokines (Rebola et al., 2011), purines (Pascual et al., 2012; George et al., 2015), glutamate and D-serine (Scianni et al., 2013), NO (Zhan et al., 2014) or BDNF (Gomes et al., 2013; Parkhurst et al., 2013). The importance of this bi-directional communication between synapses and microglia is best heralded by the synaptic dysfunction observed upon genetic manipulation of microglia function (Roumier et al., 2004; Costello et al., 2011; Hoshiko et al., 2012), which can be direct or involve astrocytes (Pascual et al., 2012). Thus, microglia are critical for the dynamic synaptic carving that is essential to entrain adaptive brain function (Paolicelli et al., 2011; Ji et al., 2013; Cristovão et al., 2014; Zhan et al., 2014).

## Dysfunction of Glial Cells and Depression

**Figure 1:**
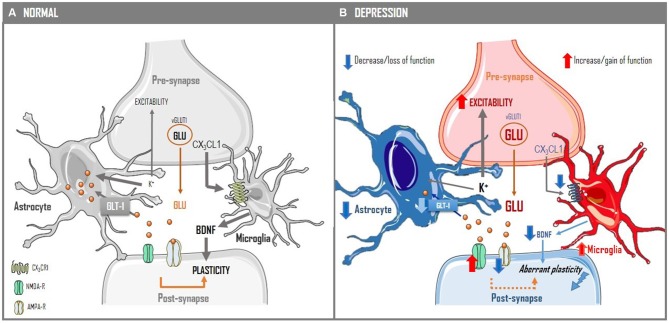
**Modifications of the quad-partite synapse in depression.** The processing of information in synapses is not only defined by neurons, but also by glia cells, namely by astrocytes, which enwrap synapses, and microglia, which dynamically interact with synapses in an activity-dependent manner **(A)**. Thus, amongst other roles, astrocytes regulate both K^+^ and the basal levels of glutamate, defining the basal excitability of neurons; also the extracellular levels of glutamate contribute to define the density of NMDA and AMPA receptors in the plasma membrane of the post-synaptic compartment, which trigger and sustain alterations of synaptic plasticity, respectively. The excitability of neurons, their set-up of plasma membrane glutamate receptors, namely of NMDA receptors, and the neurotrophins support provided by glial cells are critical to allow the implementation of adequate synaptic plasticity traits, i.e., an appropriate encoding of both long-term potentiation (LTP) and depression (LTD) according to specific patterns of incoming information. In depression **(B)** there is a modification of information processing in excitatory synapses in frontolimbic area: there is modification of astrocytes, typified by hypertrophic cell bodies and decreased complexity and activity of phylopodia, resulting in an hypofunction of astrocytes and a decreased ability to buffer K^+^ and to clear extracellular glutamate; this leads to increased excitability, corresponding to greater noise in the circuit, and to an imbalance of NMDA and AMPA receptors, with an increased availability of NMDA receptors in dendrites. Additionally, there is a decreased signaling through the master chemokine CX3CL1 in microglia, which is associated (through mechanisms still undefined) to a shift of microglia towards a more pro-inflammatory profile associated with decreased microglia-derived BDNF release. These alterations of the quad-partite synapse, typified by the altered inter-relations between glial cells and neurons, are associated with an aberrant plasticity, typified by a shift in the stimuli triggering LTD and LTP, which translates into inadequate encoding of information in frontocortical circuits.

Numerous lines of evidence support the contention that a modification of astrocytes in frontolimbic regions is associated with depression (Altshuler et al., 2010; Rajkowska and Stockmeier, 2013; Peng et al., 2015). Most studies analyzing post-mortem brain samples from adult individuals with major depressive disorder or suicide completers concur to conclude that there is a decreased number of astrocytic-like elements in frontolimbic structures (Ongür et al., 1998; Rajkowska et al., 1999; Cotter et al., 2002; Medina et al., 2015; Nagy et al., 2015; Torres-Platas et al., 2015). This is paralleled by an alteration of astrocytic morphology, typified by hypertrophic cell bodies (Rajkowska et al., 1999; Cotter et al., 2002; Torres-Platas et al., 2011, 2015), and a modification in frontolimbic regions of the density of different astrocytic markers, such as GFAP (Miguel-Hidalgo et al., 2000; Si et al., 2004; Schlicht et al., 2007; Gittins and Harrison, 2011), connexins (Ernst et al., 2011; Sun et al., 2012; Miguel-Hidalgo et al., 2014), aquaporin-4 (Rajkowska et al., 2013), GLT-1 and glutamine synthase (Choudary et al., 2005; Sequeira et al., 2009; Miguel-Hidalgo et al., 2010) and an increased release of S100β (e.g., Grabe et al., 2001; Schroeter et al., 2008). A causal relation between astrocytic dysfunction and depression is provided by animal studies showing that the selective destruction of frontocortical astrocytes with the gliotoxin L-α-aminoadipic acid is sufficient to trigger a depressive-like phenotype (Banasr and Duman, 2008); likewise, a depressive-like phenotype also emerges upon functional inhibition of astrocytes, such as upon downregulating synaptobrevin-2 thus blunting astrocytic vesicular release (Cao et al., 2013), upon altering connexin-mediated gap-junctions (Sun et al., 2012), upon knocking out IP3-receptor type-2 (Cao et al., 2013) or aquaporin-4 (Kong et al., 2014) or inhibiting astrocytic glutamate transporters (Bechtholt-Gompf et al., 2010; John et al., 2012). Notably, astrocytic function is affected by the signaling systems assumed as traditional culprits of depression, namely glucocorticoids (Yin et al., 2013), BDNF (Ye et al., 2011; Liu et al., 2015), serotonin (Hertz et al., 2015), noradrenaline (Madrigal et al., 2009; Pankratov and Lalo, 2015) or dopamine (Shao et al., 2013). Furthermore, treatments alleviating depressive symptoms can recover astrocytic function, such as SSRIs (Czéh et al., 2006; Schipke et al., 2011) or electroconvulsive shock (Iwata et al., 2011) and some are even critically dependent on astrocytic function, such as fluoxetine (Kong et al., 2009) or deep brain stimulation (Etiévant et al., 2015). Altogether, these observations indicate that a decreased astrocytic function in frontolimbic regions is necessary and sufficient for the emergence of depressive symptoms (Figure 1). This suggests a scenario where a defective astrocyte function initially hampers synaptic plasticity, which then evolves into neuronal loss at advanced phases of depressive disorders.

Depression is also tightly associated with alterations of microglia and inflammation (Yirmiya et al., 2015; Figure 1). Thus, depressive patients exhibit increased levels of peripheral inflammatory markers (Raison et al., 2006; Howren et al., 2009); conversely, bolstering inflammation triggers a sickness behavior reminiscent of depression (Dantzer et al., 2008) and patients with inflammatory and autoimmune diseases often experience depression (Kiecolt-Glaser et al., 2015). Indeed, most studies converge to propose that microglia are morphologically altered in frontolimbic regions of depressed patients or suicide completers (Steiner et al., 2008; Schnieder et al., 2014; Torres-Platas et al., 2014; Setiawan et al., 2015). Likewise, repeated stress in rodents also triggers microglia dystrophy (Kreisel et al., 2014; Milior et al., 2015; Ślusarczyk et al., 2015) and the manipulation of microglia function, altering its dynamic (Kreisel et al., 2014) or hampering microglia-neuron communication via the CX3CR1-fractalkine pathway (Corona et al., 2010; Milior et al., 2015), alters stress responsiveness and depressive-like behavior. Furthermore, microglia function is affected by all the signaling systems traditionally associated with depression, namely glucocorticoids (Ros-Bernal et al., 2011), BDNF (Gomes et al., 2013), serotonin (Müller and Schwarz, 2007; Krabbe et al., 2012), noradrenaline or dopamine (Färber et al., 2005). Further highlighting the role of microglia in depression are the observations that antidepressants like ketamine (Walker et al., 2013), fluoxetine (Chung et al., 2011) or citalopram (Su et al., 2015) regulate microglia function and minocycline, an inhibitor of microglia, simultaneously recovers microglia function and emotional impairments (Hinwood et al., 2013). Thus, the available evidence indicates that microglia dysfunction is a core event in depression (Figure 1), affecting synaptic plasticity either directly (Paolicelli et al., 2011; Zhan et al., 2014) or indirectly through its ability to control astrocytic function (Pascual et al., 2012).

## Integrative Role of Purines in the Quad-Partite Synapse to Control Depression

**Figure 2:**
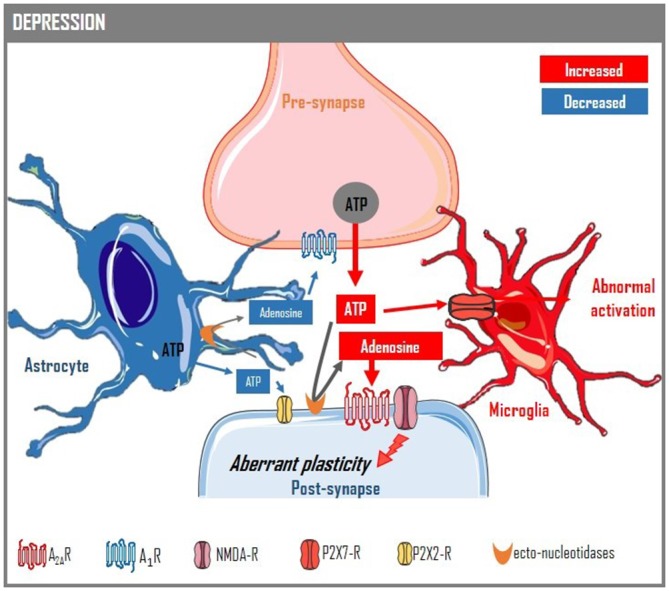
**Purine-based control of glia-neuron bidirectional communication in depression.** Associated with astrocytic hypofunction in depression, there is a lower release of ATP and adenosine from astrocytes (Cao et al., 2013; Hines et al., 2013). This causes a deficient astrocyte-to-neuron activation of P2X2 (ATP) receptors (Cao et al., 2013) and a deficient activation of inhibitory adenosine A_1_ receptors in neurons (Serchov et al., 2015), which density decreases upon chronic stress (Cunha et al., 2006; Kaster et al., 2015). In parallel, there is an increased excitability (increased noise), which bolsters the synaptic release of ATP (Cunha et al., 1996), an up-regulation of synaptic adenosine A_2A_ receptors (Cunha et al., 2006; Batalha et al., 2013; Kaster et al., 2015), which is associated with aberrant plasticity (Li et al., 2015), and an up-regulation of microglia P2X7 receptors, which contributes to microglia hyper-responsiveness upon depression (Stokes et al., 2015). This illustrates the role of the purinergic system in the control of the homeostasis of the quad-partite synapse and shows that maladaptive changes in the purine neuromodulation system occur upon depression that can be exploited therapeutically, such as increasing the release of ATP and adenosine from astrocytes, decreasing the neuronal activation of A_2A_ receptor or bolstering the neuronal activation of A_1_ receptors or inhibiting microglia P2X7 receptors.

In view of the key role of aberrant synaptic plasticity involving neuronal, astrocytic and microglia dysfunction (Figure 1), therapeutic strategies to manage depression should ideally target systems dedicated to the control of neuron-glia bidirectional communication. Purines operate one such system through the action of ATP and adenosine (Figure 2). ATP is released in a controlled manner from synaptic terminals, astrocytes and microglia and it is a documented signal to control astrogliosis, microglia dynamics and reactivity and synaptic transmission through ionotropic P2X1–7 and metabotropic P2Y1–13 receptors (reviewed in Rodrigues et al., 2015). Adenosine can be formed from the catabolism of extracellular ATP (Augusto et al., 2013) by ectonucleotidases located in synapses (Cunha, 2001) or released as such through bidirectional nucleoside transporters in synapses (Pinto-Duarte et al., 2005). Adenosine mainly activates inhibitory A_1_ and facilitatory A_2A_ receptors (Fredholm et al., 2005) that act neuronally to control synaptic transmission and plasticity (Cunha, 2008) and also control astrocytic (van Calker and Biber, 2005; Matos et al., 2013, 2015) and microglia function (Rebola et al., 2011; Luongo et al., 2014; George et al., 2015).

Notably, both ATP and adenosine signaling have been implicated in the control of depressive conditions (Figure 2). Thus, a deficient astrocyte-derived ATP release providing an insufficient P2X2 receptor-mediated neuronal tonus was identified in chronically stressed mice (Cao et al., 2013); also, the prevention of excessive P2X7 receptor activation in glial cells ameliorates depressive-like conditions (Stokes et al., 2015) and P2X7 receptor polymorphisms lead to vulnerability to mood disorders (Bennett, 2007). The case for an involvement of adenosine is more robust. Thus, epidemiological studies show an inverse relation between the intake of moderate amounts of caffeine (an adenosine receptor antagonist) and the incidence of depression (Lucas et al., 2011) and suicide (Lucas et al., 2014). Accordingly, adenosine A_2A_ receptors are up-regulated in animal models of chronic stress and polymorphisms of A_2A_ receptors are associated with emotional disturbances (reviewed in Cunha et al., 2008), their over-expression triggers emotional dysfunction (Coelho et al., 2014) and their blockade prevents chronic stress-induced emotional dysfunction (Kaster et al., 2015). Additionally, there is a hypofunction of neuronal A_1_ receptors due to decrease astrocyte-derived adenosine (Hines et al., 2013), which compensation with various antidepressant treatments can revert depressive-like behavior (Etiévant et al., 2015; Serchov et al., 2015).

This compilation of evidences illustrates the relevance of the purinergic signaling in the control of neuron-glia bidirectional communication and its therapeutic potential in the normalization of aberrant synaptic processing in frontolimbic circuits upon depression. Based on the available information, the most promising strategy is a multi-target approach, based on the increase of astrocytic release of purines (both ATP and adenosine to activate A_1_ receptors) coupled to antagonists of P2X7 and of A_2A_ receptors (Figure 2).

## Author Contributions

RAC planned and organized the review; all others contributed with valuable suggestions, partial writting and editing of the review.

## Conflict of Interest Statement

The authors declare that the research was conducted in the absence of any commercial or financial relationships that could be construed as a potential conflict of interest.
